# The effects of gender-affirming hormone therapy on myocardial, hepatic, pancreatic lipid content, body fat distribution and other cardiometabolic risk factors: A magnetic resonance-based study in transgender individuals

**DOI:** 10.1016/j.jcte.2024.100379

**Published:** 2024-12-06

**Authors:** Dorota Sluková, Carola Deischinger, Ivica Just, Ulrike Kaufmann, Siegfried Trattnig, Martin Krššák, Lana Kosi-Trebotic, Juergen Harreiter, Alexandra Kautzky-Willer

**Affiliations:** a Department of Internal Medicine III, Clinical Division of Endocrinology and Metabolism, Gender Medicine Unit, Medical University of Vienna, General Hospital Vienna, Waehringer Guertel 18–20, 1090 Vienna, Austria; b High Field MR Centre of Excellence, Department of Biomedical Imaging and Image-guided Therapy, Medical University of Vienna, Lazarettgasse 14, 1090 Vienna, Austria; c Department of Obstetrics and Gynaecology, Clinical Division of Gynaecologic Endocrinology and Reproductive Medicine, General Hospital Vienna, Waehringer Guertel 18-20, 1090 Vienna, Austria; d Department of Medicine, Landesklinikum Scheibbs, Austria

**Keywords:** Gender-affirming hormone therapy, Intraorgan lipid content, Adipose tissue distribution, Glucose homeostasis

## Abstract

**Purpose:**

We aimed to assess the changes in body fat distribution, intraorgan lipid accumulation, and cardiometabolic risk factors after 6 months of gender-affirming hormone therapy (GAHT) in transgender men (TM) and transgender women (TW).

**Methods:**

Conducted at the Medical University of Vienna between 2019 and 2022, the study included 15 TW and 20 TM. We conducted magnetic resonance imaging and spectroscopy to determine the visceral (VAT) and subcutaneous adipose tissue (SAT) amounts, the VAT/SAT ratio, and the intraorgan lipid content (liver, pancreas, myocardium), bloodwork, and an oral glucose tolerance test at baseline and after 6 months of GAHT.

**Results:**

Pancreatic, hepatic, and intramyocardial lipid contents did not significantly change in either group after 6 months of GAHT. In TW, VAT/SAT ratio decreased significantly from baseline 0,930 (IQR 0,649–1,287) to 0,758 (IQR 0,424–0,900; p = 0,011) after 6 months of GAHT. The updated homeostatic model assessment for insulin sensitivity (HOMA2-%S) significantly decreased from 83,03 % (±31,11) to 64,27 % (±18,01; p = 0,047), indicating decreased insulin sensitivity, while the updated homeostatic model assessment for β-cell function (HOMA2-%β) increased (from 128,11 % (±35,80) to 156,80 % (±39,49); p = 0,020) in TW after 6 months of GAHT. In TM, there were no changes in glucose metabolism parameters except for an increase in HbA_1c_ (5,1% (±0,3) vs 5,3% (±0,4), p = 0,001).

**Conclusions:**

6 months of GAHT were not associated with statistically significant changes in myocardial, hepatic, or pancreatic lipid content. Short-term GAHT led to a marked body fat redistribution with a significant decrease in the VAT/SAT ratio in TW.

## Introduction

Sex hormones play an important role in adipose tissue distribution, the development of prediabetes and type 2 diabetes mellitus (T2DM), and other cardiovascular risk factors between cisgender men and cisgender women [1], [2], [3]. Thus, it is rational to assume that changes in sex hormone profiles due to gender-affirming hormone therapy (GAHT) might also lead to changes in adipose tissue distribution and glucose tolerance. However, there is still a lack of conclusive evidence on the changes brought on by GAHT, especially concerning intraorgan lipid content.

Cisgender women generally show less ectopic lipid accumulation than cisgender men [2], [4], [5], [6], [7], [8]. To our knowledge, no studies to date have concerned themselves with changes in myocardial and pancreatic lipid content in transgender people under GAHT, and only one study observed a decrease in hepatic lipid content in transgender women and an increase in hepatic lipid content in transgender men after 1 year of GAHT including GnRH analogs [9].

Regional adipose tissue distribution shows a typical dimorphism between cisgender women and cisgender men – visceral adipose tissue (VAT) accumulation is more pronounced in cisgender men, and is associated with a higher risk of cardiovascular and metabolic diseases compared to subcutaneous adipose tissue (SAT) accumulation in the lower body, which is more prominent in cisgender women [1], [7], [10], [11]. Additionally, in cisgender men, abdominal subcutaneous and visceral adipose tissue both seem to be similarly associated with insulin resistance, whereas insulin resistance in cisgender women seems to be particularly associated with the accumulation of visceral adipose tissue in the abdominal region [3].

During GAHT, transgender people tend to gain weight and experience a change in body composition − transgender women gain fat mass and lose lean mass, whereas the opposite happens in transgender men [12], [13], [14], [15], [16], [17], [18]. These results are relatively consistent across studies with different measurement methods (bioimpedance, magnetic resonance, dual-energy X-ray absorptiometry (DXA)) and different treatment protocols [16], [17]. However, it is important to note that previous studies often included medications that are no longer used in contemporary GAHT protocols, such as ethinylestradiol [19], [20], [21].

GAHT also impacts lipid profiles and glucose metabolism. In transgender women, feminizing hormone therapy leads to generally favorable lipid profile changes, resembling premenopausal cisgender women, with lower LDL cholesterol and triglyceride levels compared to pre-GAHT [10], [22], [23], [24], [25], [26], [27]. Additionally, GAHT is associated with no to only slightly detrimental effects on glucose homeostasis and insulin resistance in transgender women, generally observed as an increase of fasting insulin and the HOMA-IR, without significant glucose level changes [17], [22], [27], [28]. In transgender men, the data is somewhat inconsistent − some studies report elevations of triglycerides and decreases of HDL cholesterol, while others also note an increase of LDL cholesterol compared to baseline [22], [23], [25], [26], [27], [29], [30]. Studies into GAHT effects on glucose metabolism in transgender men note either no changes or slight improvements in insulin resistance without changes in glucose levels [17], [22], [28].

This study aimed to investigate short-term changes in intraorgan lipid content, the subcutaneous and visceral adipose tissue distribution, as well as changes in lipid and glucose homeostasis in transgender individuals after 6 months of GAHT. We focused on localized MR-spectroscopy and imaging to determine abdominal VAT/SAT as well as the lipid content of the myocardium, liver, and pancreas, which has not been sufficiently investigated in people under GAHT so far.

## Materials and methods

### Study design

This monocentric longitudinal study was conducted at the Department of Endocrinology and Metabolism and the Highfield MR Centre of Excellence at the Medical University of Vienna between 2019 and 2022. The study adhered to the Declaration of Helsinki and was approved by the local ethics committee. Before participating in the study, all participants provided written informed consent after receiving thorough information.

### Study participants

The study included 15 transgender women (assigned male at birth) and 20 transgender men (assigned female at birth). Two study visits were conducted for each participant: the first before the initiation of gender-affirming hormone treatment (GAHT), and the second visit 6 months after treatment started. GAHT was indicated and administered entirely separately from the study, with treatment regimens and duration of the treatment not influenced by participation in the study. Transgender women included in the study used either transdermal or oral estrogen medication, usually in combination with cyproterone acetate; transgender men received intramuscular or transdermal testosterone. Due to the limited sample size, we were unable to stratify for the mode of application. Such stratification was further complicated by patients not necessarily adhering to one mode of application during the treatment.

The study included transgender participants diagnosed with “gender dysphoria in adolescents and adults”, classified under the number 302.85 in the 5th edition of the Diagnostic and Statistical Manual of Mental Disorders (DSM-V) who were yet to begin GAHT and were in generally good health. Exclusion criteria included severe diseases (neurological and/or internal), significant abnormalities upon routine screenings or physical examination, pregnancy, general contraindications for magnetic resonance exams (i.e., non-MR-conditional devices or implants), current substance abuse, and/or non-compliance with the study protocol.

### Measurements and methods

All measurements were performed at the outpatient clinic of the Department of Endocrinology and Metabolism and at the High Field MR Centre of Excellence after an overnight fast of at least 8 h, with all patients undergoing the same set of examinations on both study visits.

First, the participants underwent a magnetic resonance imaging and spectroscopy exam (MRI/MRS) in a 3-Tesla magnetic resonance device Magnetom Prisma Fit (Siemens Healthineers, Erlangen, Germany) to measure the lipid content in the myocardial, hepatic, and pancreatic tissues, and the distribution of VAT and SAT. The MRI and MRS measurements were performed in accordance with established methods at the High Field MR Centre of the Medical University of Vienna. Myocardial lipid content was measured with electrocardiogram-gated ^1^H-MR spectroscopy evaluating the spectral signals acquired from the interventricular septum, similar to the exams conducted in previous studies [31], [32]. The hepatic lipid content was determined by short echo time single-voxel MRS, whereas lipid content in the pancreas was quantified by multi-echo Dixon imaging sequences delivering fat fraction images; both of the measurements were done following previously published protocols [32], [33], [34]. Additionally, the amounts of SAT and VAT were measured using magnetic resonance imaging at the height of the intervertebral disc between L2/L3 in an axial slice in T1-weighted images, enabling the calculation of the visceral to subcutaneous adipose tissue ratio.

Following the MR exam, the patients underwent extensive bloodwork to assess the levels of hormones (luteinizing hormone, follicle-stimulating hormone, 17-β-estradiol, testosterone, sex hormone-binding globulin, androstenedione, dehydroepiandrosterone sulfate, 25-hydroxyvitamin D_3_) and baseline metabolic parameters (fasting glucose, fasting insulin, parameters of lipid metabolism (total cholesterol, low-density lipoprotein cholesterol, high-density lipoprotein cholesterol, triglycerides, glycosylated hemoglobin (HbA_1c_) and a 75 g oral glucose tolerance test (OGTT). During the 2-hour OGTT, blood was drawn through an intravenous cannula placed in the cubital vein at 30-minute intervals (i.e., at baseline, after 30, 60, 90, and 120 min after glucose ingestion) to assess the time course of glucose, insulin, and c-peptide and to enable the subsequent calculation of surrogate markers of insulin sensitivity. We utilized the updated homeostasis model assessment for insulin sensitivity (HOMA2-%S) and the homeostasis model assessment for β-cell function (HOMA2-%β), which were calculated using the HOMA Calculator Version 2.2.3., available online at https://www.rdm.ox.ac.uk/about/our-clinical-facilities-and-mrc-units/DTU/software/homa to assess the insulin sensitivity and insulin secretion.

The abdominal circumference was measured at the lower border of the rib cage, whereas weight was measured with an electronic scale (SECA 877/888) in light clothing.

All laboratory assessments were conducted at the ISO 9001 central laboratory of the Clinical Institute for Laboratory Medicine at Vienna General Hospital, the exact methods can be found at the website www.kilm.at.

### Statistical analysis

The data was tested for normal distribution by the Shapiro-Wilk test. In the case of data following a normal distribution, the results are presented as mean (±standard deviation), or as median (interquartile range) if the data did not follow normal distribution. In order to assess the differences between the baseline values and values after 6 months of GAHT, the data was analyzed using the Wilcoxon signed-rank test. Possible correlations between the variables of interest were calculated using Spearman's rank correlation coefficient. Correlations were calculated for the values at follow-up. The level of significance was set at α = 0,05. The statistical analysis was done using IBM SPSS Statistics software version 29.0.0.0 (IBM, New York, USA).

## Results

In 2 individuals, we were unable to complete the full MR examination due to physical proportions (1 TW) and claustrophobia (1 TM).

The mean age of TW participating in our study at baseline was 35,33 years (±11,00), while for TM, the mean age at the beginning of the study was 23,80 years (±5,16).

As expected, the hormone profiles of transgender persons after 6 months of GAHT changed to resemble the hormone profiles of the cisgender people of their affirmed gender. Detailed hormone profiles at baseline and after 6 months of GAHT can be found in Table 1.

**Table 1:** Hormone panels in transgender men and transgender women at baseline and after 6 months of gender-affirming hormone therapy (GAHT). The data is given as mean ± standard deviation (SD) if normally distributed, or as median and interquartile range (IQR) if not normally distributed. LH = luteinizing hormone, FSH = follicle-stimulating hormone, bioavailable T. = bioavailable testosterone, SHBG = sex hormone-binding globulin, DHEAS = dehydroepiandrosterone sulfate, 25-OH-Vitamin D = 25-hydroxy-Vitamin D.

Mean (±standard deviation) or Median (interquartile range)	Transgender women		Transgender men	
Hormone panel	Baseline	After 6 months	Baseline	After 6 months
LH (mIU/ml)	4,1 (2,8–5,9)	0,3 (0,2–0,7)	6,5 (5,5–9,0)	3,8 (2,6–9,4)
FSH (mIU/ml)	3,1 (2,0–4,2)	0,3 (0,2–0,9)	4,3 (3,7–5,9)	4,7 (2,3–6,3)
Prolactin (ng/ml)	9,9 (8,0–16,5)	26,1 (16,8–45,7)	16,4 (11,0––25,3)	14,0 (9,6–19,3)
Progesterone (ng/ml)	0,15 (0,04–0,27)	0,19 (0,07–3,26)	0,34 (0,27–5,16)	0,26 (0,11–0,92)
17-β-estradiol (pg/ml)	33,0 (21,0–53,0)	124,0 (68,0–204,0)	68,5 (45,8– 135,0)	52,0 (39,8–98,8)
Testosterone (ng/ml)	4,40 (4,22–6,00)	0,20 (0,1–0,41)	0,36 (0,29–0,50)	7,05 (3,52–9,36)
Bioavailable T. (ng/ml)	2,47 (1,73–2,71)	0,06 (0,04–0,14)	0,12 (0,10–0,18)	3,38 (1,14–5,69)
SHBG (nmol/l)	38,0 (±16,1)	48,9 (±20,1)	48,5 (32,6–65,8)	26,7 (22,6–44,5)
Androstenedione (ng/ml)	1,26 (±0,56)	0,88 (±0,43)	1,80 (±0,69)	2,47 (±0,97)
DHEAS (μg/ml)	2,76 (±0,93)	2,63 (±1,29)	2,92 (±1,07)	3,11 (±1,16)
25-OH-Vitamin D (nmol/l)	46,7 (±21,2)	56,2 (±21,2)	49,4 (±25,0)	54,4 (±25,9)

**Fig. 1:**
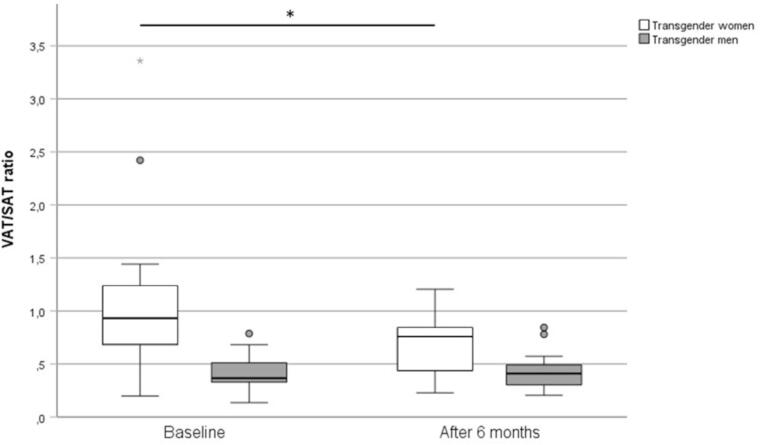
Boxplots of the visceral to subcutaneous adipose tissue ratio (VAT/SAT ratio) at baseline and after 6 months. The asterisk above the solid line symbolizes a significant change in the VAT/SAT ratio in transgender women after 6 months of GAHT at the significance level α = 0,05.

In transgender women, we did not detect statistically significant differences in organ lipid percentages in the MR-based measurements after 6 months of feminizing GAHT compared to baseline, despite the median hepatic and myocardial lipid contents decreasing numerically and the mean pancreatic lipid content increasing numerically. The mean subcutaneous fat area increased and the mean visceral fat area decreased numerically, which was in line with the observed significant decrease in the VAT/SAT ratio (Fig. 1). However, we did not observe any significant changes in weight, BMI, or abdominal circumference after 6 months of feminizing GAHT.

In transgender women, mean concentrations of total and LDL cholesterol decreased significantly, while the concentrations of triglycerides increased minimally. In addition, we observed a significant increase in the HOMA2-%β index accompanied by a significant decrease in the HOMA2-%S after 6 months of feminizing GAHT. The detailed results for the cohort of transgender women can be found in Table 2.

**Table 2:** Outcome measures in transgender women at baseline and after 6 months of gender-affirming hormone therapy. The data is given as mean ± standard deviation (SD) if normally distributed, or as median and interquartile range (IQR) if not normally distributed. MR = magnetic resonance. VAT/SAT ratio = ratio of visceral adipose tissue to subcutaneous adipose tissue. SAT = subcutaneous adipose tissue. VAT = visceral adipose tissue. LDL-C = low-density lipoprotein cholesterol. HDL-C = high-density lipoprotein cholesterol. HbA_1c_ = glycated hemoglobin. HOMA2-%S = updated homeostatic model assessment for insulin sensitivity. HOMA2-%β = updated homeostatic model assessment for β-cell function. BMI = body mass index. P-values marked with an asterisk indicate statistical significance at the level of <0,05.

Transgender women			
Primary endpoints MR parameters	Baseline	After 6 months	p-value
Myocardial lipid content (%)	0,45 (0,19–0,83)	0,40 (0,29–0,62)	0,754
Hepatic lipid content (%)	0,81 (0,40–1,86)	0,67 (0,41–1,44)	0,126
Pancreatic lipid content (%)	5,54 (±2,80)	6,66 (±3,11)	0,071
VAT/SAT ratio	0,930 (0,649–1,287)	0,758 (0,424–0,900)	0,011*
SAT area (mm^2^)	13,730 (±8544)	15,038 (±9323)	0,096
VAT area (mm^2^)	13,983 (±9909)	10,830 (±8042)	0,064

**Secondary endpoints** **Metabolic parameters**			
Total cholesterol (mg/dl)	161 (±48)	149 (±38)	0,020*
LDL-C (mg/dl)	93 (±41)	83 (±34)	0,020*
HDL-C (mg/dl)	47 (42–60)	43 (39–49)	0,462
Triglycerides (mg/dl)	82 (60–117)	85 (57–97)	0,035*
Fasting glucose (mg/dl)	87 (±7)	85 (±6)	0,054
Glucose after 120 min (mg/dl)	109 (±21)	106 (±23)	0,532
Fasting insulin (μIU/ml)	10,8 (±4,2)	13,3 (±4,0)	0,069
Insulin after 120 min (μIU/ml)	59,3 (30,3–85,2)	65,6 (50,3–82,4)	0,427
HbA_1c_ (%)	4,9 (±0,4)	4,9 (±0,3)	0,318
HOMA2-%S	83,03 (±31,11)	64,27 (±18,01)	0,047
HOMA2-%β	128,11 (±35,80)	156,80 (±39,49)	0,020*

**Secondary endpoints** **Anthropometric parameters**			
Weight (kg)	80,0 (67,0–87,5)	79,5 (70,7–91,5)	0,529
BMI (kg/m2)	23,60 (22,60–27,13)	24,36 (22,57–26,17)	0,463
Abdominal circumference (cm)	85,0 (78,0–94,0)	87,5 (81,0–93,0)	0,209

In this cohort, levels of estradiol at follow-up did not correlate with any of the MR- and metabolic parameters. On the other hand, testosterone at follow-up significantly negatively correlated with the amount of VAT at follow-up (r_ρ_ = -0,566, p = 0,035). Additionally, the level of testosterone was also associated with myocardial lipid content (r_ρ_ *=* 0,696, p = 0,006).

In transgender men, the MR-based measurements of intraorgan lipid percentages did not show any significantly changes after 6 months of testosterone treatment, albeit myocardial lipid content showed a decreasing tendency. In transgender men, we observed a tendency towards an increase of VAT, which did not reach statistical significance. There were no significant changes of SAT or the VAT/SAT ratio after 6 months of GAHT in this cohort (Fig. 1). However, we observed significant increases in weight, BMI, and abdominal circumference after 6 months of masculinizing hormone treatment with testosterone.

We did not detect any significant changes in lipid profiles after 6 months of masculinizing GAHT. No changes in fasting or postprandial glucose and insulin, as well as in the calculated indices for insulin sensitivity and β-cell function were detected in transgender men. Nonetheless, transgender men exhibited a significant increase in HbA_1c_ after 6 months of testosterone treatment. The detailed results for the cohort of transgender men can be found in Table 3.

**Table 3:** Outcome measures in transgender men at baseline and after 6 months of gender-affirming hormone therapy. The data is given as mean ± standard deviation (SD) if normally distributed, or as median and interquartile range (IQR) if not normally distributed. MR = magnetic resonance. VAT/SAT ratio = ratio of visceral adipose tissue to subcutaneous adipose tissue. SAT = subcutaneous adipose tissue. VAT = visceral adipose tissue. LDL-C = low-density lipoprotein cholesterol. HDL-C = high-density lipoprotein cholesterol. HbA1c = glycated hemoglobin. HOMA2-%S = updated homeostatic model assessment for insulin sensitivity. HOMA2-%β = updated homeostatic model assessment for β-cell function. BMI = body mass index. P-values marked with an asterisk indicate statistical significance at the level of <0,05.

Transgender men			
Primary endpoints MR parameters	Baseline	After 6 months	p-value
Myocardial lipid content (%)	0,61 (0,30–0,99)	0,29 (0,22–0,51)	0,084
Hepatic lipid content (%)	0,42 (0,25–1,10)	0,53 (0,34–0,78)	0,965
Pancreatic lipid content (%)	5,60 (3,88–7,54)	5,97 (3,94–7,16)	0,795
VAT/SAT ratio	0,367 (0,325–0,512)	0,409 (0,298–0,505)	0,433
SAT area (mm^2^)	16,714 (8908–23638)	16,390 (8974–27825)	0,658
VAT area (mm^2^)	5999 (3755–11460)	6840 (3769–10320)	0,171

**Secondary endpoints** **Metabolic parameters**			
Total cholesterol (mg/dl)	163 (±27)	156 (±34)	0,888
LDLC (mg/dl)	92 (±26)	98 (±35)	0,365
HDLC (mg/dl)	52 (±13)	48 (±12)	0,121
Triglycerides (mg/dl)	78 (54–140)	80 (63–142)	0,380
Fasting glucose (mg/dl)	82 (±8)	81 (±10)	0,506
Glucose after 120 min (mg/dl)	102 (85–131)	115 (91–129)	0,538
Fasting insulin (μIU/ml)	10,3 (±4,7)	10,3 (±6,0)	0,970
Insulin after 120 min (μIU/ml)	73,9 (40,6–103,2)	94,4 (49,0–114,8)	0,156
HbA_1c_ (%)	5,1 (±0,3)	5,3 (±0,4)	0,001*
HOMA2-%S	81,40 (60,10––122,48)	84,35 (59,60–146,45)	0,940
HOMA2-%β	140,29 (±48,32)	140,29 (±54,07)	0,940

**Secondary endpoints Anthropometric parameters**			
Weight (kg)	67,90 (57,15–76,83)	73,65 (61,25–82,28)	0,015*
BMI (kg/m2)	25,60 (±5,35)	26,45 (±4,96)	0,015*
Abdominal circumference (cm)	85,5 (74,0–92,8)	88,7 (75,1–100,0)	0,021*

In transgender men, we observed a negative correlation between estradiol levels and pancreatic lipid content at follow-up (r_ρ_ = −0,528, p = 0,024), while testosterone did not significantly correlate with any of the MR-based parameters. In the case of metabolic parameters, testosterone was associated with the HOMA2-%S values (r_ρ_ = 0,486, p = 0,030).

## Discussion

Our study focused on examining the changes in MR-measured lipid content in the liver, pancreas and myocardium, adipose tissue distribution, and other cardiometabolic risk factors in transgender women and transgender men before initiating GAHT and after 6 months of the treatment.

Regarding changes in intraorgan lipid content, we did not detect any statistically significant changes in any organs in either cohort. Despite the results not being statistically significant, there was a trend towards an increase in pancreatic lipid content and towards a decrease in hepatic lipid content in transgender women, while transgender men exhibited a tendency towards a decrease in myocardial lipid content.

**Fig. 2:**
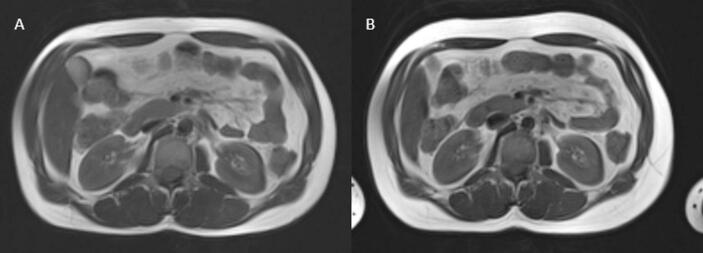
Differences in VAT and SAT distribution at the level of L2/L3 in a transgender woman before (A) and after (B) 6 months of gender affirming hormone therapy. Overall, in transgender women, we observed a statistically significant decrease in the VAT/SAT ratio after 6 months of feminizing GAHT. Additionally, we observed a tendency towards an increase of SAT and a decrease of VAT in this cohort.

In transgender women, we observed significant changes in abdominal adipose tissue distribution − in our study, the VAT/SAT ratio measured at a cross-sectional slice at the level of the L2/L3 vertebrae decreased significantly (Fig. 1). This significant decrease of the VAT/SAT ratio seems to be the consequence of a less significant increase in SAT area and a less significant decrease in the VAT area in the transgender female individuals, illustrated by Fig. 2. In cisgender persons, more pronounced adipose tissue storage in the subcutaneous than in the visceral compartment is more common in premenopausal cisgender women than cisgender men, and seems to be less detrimental for cardiometabolic health than the opposite [1], [7], [11]. Contrastingly, previously published literature noted increases in both SAT and VAT in transgender women using GAHT [19], [20], [21], [35]. Additionally, we did not observe the changes of weight or abdominal circumference noted in previous reports, which might be attributed to the reciprocal changes of VAT and SAT [15], [16]. However, the results of these studies are not directly comparable due to different follow-up duration and differing GAHT protocols used [20], [21].

**Fig. 3:**
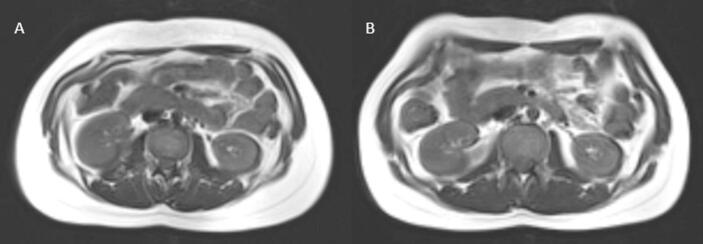
Differences in VAT and SAT distribution at the level of L2/L3 in a transgender man before (A) and after (B) 6 months of gender affirming hormone therapy. In transgender men, we observed a tendency towards an increase of VAT, whereas we did not observe any changes of SAT or the VAT/SAT ratio after 6 months of GAHT.

In transgender men, we observed a tendency towards an increase in abdominal visceral adipose tissue (Fig. 3), which was not statistically significant. Such tendency has also been reported in previous studies [9], [19], [20], [21]. However, this was not accompanied by significant changes neither in the VAT/SAT ratio, nor in the SAT area, which is in contrast with earlier research [9], [19], [20], [21]. The results presented by Tebbens et al. [9] demonstrated a decrease of the ratio of subcutaneous to visceral adipose tissue accompanied by an increase in VAT after 12 months, while other MR-based studies with a 1-year follow up period also note a decrease of subcutaneous abdominal fat [9], [19], [20], [21]. A direct comparison between the results of those studies is not fully feasible due to the differing follow up length and marked differences in the treatment protocols [9], [20], [21]. Nevertheless, these results suggest that changes in adipose tissue distribution in transgender men might require a longer time to develop.

When discussing changes in body composition and fat distribution under GAHT, it is important to consider that the variability of the changes and interindividual differences in their extent is high, with a non-negligible amount of individuals not experiencing any changes at all [13], [14].

We observed a decreasing tendency of hepatic lipid content after 6 months of feminizing GAHT which was not statistically significant, whereas Tebbens et al. [9] observed a decrease of 1,55 % after 12 months in this group. Additionally, we observed a minimal numerical increase in hepatic lipid content in transgender men which was also not statistically significant, whereas Tebbens et al. [9] report a significant increase thereof by 0.83 %. The differences might be due to the shorter duration of our study and differing treatment protocols, since Tebbens et al. [9] also utilized GnRH-analogues.

To our knowledge, our study is the first concerning itself with myocardial and pancreatic lipid content in transgender individuals under GAHT. We observed an increasing tendency in pancreatic lipid content and a minimal numerical decrease in myocardial lipid content in transgender women, whereas in transgender men, we noted a tendency towards a decrease in the myocardial lipid content. Despite the interesting trends in both groups, none of the results reached statistical significance, highlighting a need for further exploration after a longer therapy duration.

We also examined the possible correlations between the sex hormone levels and the changes in MR-based and metabolic parameters at follow up. In transgender women, levels of estradiol at follow up did not correlate with any of the MR-based or metabolic parameters, while testosterone levels after 6 months of GAHT were moderately negatively associated with VAT at follow up. Additionally, testosterone in transgender women also correlated positively with myocardial lipid content, indicating that insufficiently suppressed testosterone concentrations in TW might be associated with higher levels of myocardial lipid content.

In transgender men, there was a moderate negative correlation between estradiol levels and pancreatic lipid content, indicating that higher levels of estradiol in TM might be associated with lower levels of pancreatic adiposity. In transgender men, the levels of testosterone did not significantly correlate with any of the MR-based parameters. Regarding metabolic parameters in transgender men, higher testosterone levels were moderately associated with higher insulin sensitivity quantified by the HOMA2-%S index.

The changes in lipid profiles in transgender women are in line with the expected changes with a decrease in total cholesterol and LDL-C concentrations [22], [23], [24], [25], [26], [27]. Furthermore, we also observed significant changes in the calculated indices for assessing insulin sensitivity and β-cell function. While the HOMA2-%S − quantifying the insulin sensitivity − decreased after 6 months of GAHT, the HOMA2-%β – providing an estimate of the β-cell function − increased, which indicates a worsening of insulin sensitivity with an accompanying increase of insulin production. The previously published literature on the effects of GAHT on the glucose metabolism in transgender women is somewhat conflicting, and notes changes which range from no to slight worsening of glucose metabolism status [17], [28]. We also explored the possible relationships between visceral and subcutaneous adipose tissue and the HOMA-2 indices. In transgender women, HOMA2-%β was positively associated with the amount of both SAT and VAT. On the other hand, HOMA2-%S was negatively correlated with adipose tissue amount in both compartments, with a stronger correlation with VAT. This is in line with the findings made in cisgender women, in which visceral adiposity seems to be more strongly associated with insulin resistance than subcutaneous adipose tissue [3].

In transgender men, we did not observe any significant changes in the blood lipid profiles. Despite no significant changes in glucose and insulin levels, we detected a statistically significant, albeit small, increase of HbA_1c_ value, reflecting a slightly worse long-term glucose tolerance after 6 months of testosterone GAHT. This is a finding that is in contrast with the generally no or slightly beneficial changes in glucose tolerance and insulin sensitivity that are observed in transgender men under GAHT [17], [28].

Previous studies on adipose tissue distribution often utilized ethinylestradiol (EE) as the main estrogen medication in feminizing GAHT, which is associated with significant adverse effects and therefore no longer in use [19], [20], [21]. Given contemporary treatment protocols, our study, which excludes EE, offers an updated insight into the changes in adipose tissue distribution measured by MR imaging and cardiometabolic risk factors. Furthermore, we provide a novel insight into the changes in organ lipid content of transgender individuals after 6 months of GAHT using magnetic resonance imaging and spectroscopy, which, to our knowledge, has not been explored so far apart from a single study into hepatic lipid content with a vastly different treatment protocol [9]. Follow-up studies might provide more insight into the long-term changes in organ lipid content and body fat distribution, as well as the possible clinical consequences thereof.

The differentiation between the effects of different administration methods (oral vs. transdermal estrogen, or transdermal vs. intramuscular testosterone) was impossible due to the small sample size. Additionally, in many cases, patients switch between routes of administration, further complicating a clear stratification. In all cases, the GAHT was titrated to cisgender reference ranges. Previous research into this topic has not reported any significant differences between the effects of different testosterone and estrogen formulations regarding adipose tissue distribution and body composition [18], [36]. Regarding metabolic parameters, oral estrogen seems to be connected to less favorable changes in lipid profiles compared to transdermal estrogen; in contrast, there seems to be no significant difference between testosterone formulations [18], [25], [26], [36], [37]. Generally, studies stratifying by different routes of administration are scarce; further studies with larger populations enabling a clear stratification might contribute to more precise insights into the effects of GAHT.

While our study detected interesting trends in the changes of intraorgan lipid content and adipose tissue distribution, the results must be interpreted cautiously due to the limited size of the study population and the duration of the intervention. Furthermore, we were not able to account for the psychosocial, dietary, and lifestyle circumstances accompanying the transition process, which should be more closely considered in future research.

The lack of statistical significance in some of our findings could potentially be attributed to the limited sample size, implying that our study might have been underpowered to detect more subtle changes, in the sense that a Type 2 error cannot entirely be ruled out. Further research with larger populations and longer follow-up periods is already being conducted and may reveal more definitive findings. Nonetheless, our study provides novel insights into the short-term effects of GAHT on intraorgan lipid content and adipose tissue distribution, though the immediate influence on clinical practice is limited. Future studies elucidating the effects of GAHT stratified by the mode of application could help inform the decisions taken when choosing the most suitable GAHT regime for each individual, taking into account their baseline metabolic profile and adipose tissue distribution, as well as inform clinical practice highlighting possible areas requiring closer observation during GAHT in order to provide the most suitable care for individual patients. The findings provided by our study highlight important areas for further investigation − given the typically long-term and individualized nature of GAHT, future research should involve longer study durations and broader populations also encompassing non-binary individuals and those with less conventional GAHT requirements.

## CRediT authorship contribution statement

**Dorota Sluková:** Writing – original draft, Project administration, Investigation, Formal analysis, Data curation. **Carola Deischinger:** Writing – original draft, Project administration, Investigation, Formal analysis, Data curation, Conceptualization. **Ivica Just:** Writing – review & editing, Methodology, Investigation, Data curation. **Ulrike Kaufmann:** Writing – review & editing, Supervision. **Siegfried Trattnig:** Writing – review & editing, Supervision. **Martin Krššák:** Writing – review & editing, Supervision, Methodology. **Lana Kosi-Trebotic:** Writing – review & editing, Supervision, Methodology, Funding acquisition, Conceptualization. **Juergen Harreiter:** Writing – review & editing, Supervision. **Alexandra Kautzky-Willer:** Writing – review & editing, Supervision, Conceptualization.

## Funding

The study is funded by the “Medical Scientific Fund of the Mayor of the City of Vienna”, project number 18036, awarded to Lana Kosi-Trebotic.

## Declaration of competing interest

The authors declare the following financial interests/personal relationships which may be considered as potential competing interests: Lana Kosi-Trebotic reports financial support was provided by Medical Scientific Fund of the Mayor of the City of Vienna. If there are other authors, they declare that they have no known competing financial interests or personal relationships that could have appeared to influence the work reported in this paper.
